# aDolescents gEnder surVey, rEsponsible coupLes evaluatiOn, and capacity building Project in India (DEVELOP): a study protocol

**DOI:** 10.12688/f1000research.19521.2

**Published:** 2021-03-29

**Authors:** Anand Ahankari, Mark Hayter, Clare Whitfield, Parveen Ali, Sneha Giridhari, Shruti Tambe, Pratyush Kabra, Kranti Rayamane, Pavel Ovseiko

**Affiliations:** 1 School of Health and Social Work, Faculty of Health Sciences, University of Hull, Hull, Yorkshire, HU6 7RX, UK; 2 School of Health Sciences, Faculty of Health & Medical Sciences, University of Surrey, Guildford, Surrey, GU2 7YH, UK; 3 School of Nursing and Midwifery, University of Sheffield, Sheffield, S10 2LA, UK; 4 SWISSAID, Pune, Maharashtra, 411040, India; 5 Department of Sociology, Savitribai Phule Pune University, Pune, Maharashtra, 411007, India; 6 Department of Community Medicine, Ashwini Rural Medical College, Hospital and Research Centre, Solapur, Maharashtra, 413006, India; 7 Halo Medical Foundation, Andur, Osmanabad, Maharashtra, 413603, India; 8 Radcliffe Department of Medicine, University of Oxford, Oxford, OX3 9DU, UK

**Keywords:** Gender, Maharashtra, India, Adolescent, Violence, Evaluation

## Abstract

Domestic violence and assault (DVA) against women is a serious concern in India. This affects the health and wellbeing of victims and their dependents. Published evidence has documented a variety of reasons for such violence in Indian societies, paving a pathway to design, implement, and evaluate intervention models to address this issue. DEVELOP is a research study designed by UK and Indian research teams to plan future projects to address gender-based discrimination and DVA against women and girls in India. This study protocol provides detailed information on the objectives, research methods, data collection, storage, analysis, and dissemination plans of the DEVELOP phase 1 work (2018-19). The first component is a survey of adolescent boys and girls from rural areas of the Maharashtra state of India to understand their gender equality related knowledge and beliefs. The insight gathered will be used to design interventions targeted at adolescent populations through future research and development programmes. Secondly, an evaluation of the ‘Responsible Couples’ project will be conducted to assess its success and challenges, and to inform future programme activities and strategy. The ‘Responsible Couples’ project is implemented in 40 villages of Maharashtra state to improve relationships in married couples, prevent violence against women, intervene during violence, and to provide support services for women and their family members. Research findings will be disseminated though public engagement events in India, international conferences, and peer reviewed publications. Secondly, our two key partners (SWISSAID and HMF) will benefit from such evidence to inform their on-going as well as forthcoming projects on gender equality in India.  Research findings will be also useful for local government authorities and non-government agencies striving to advance gender equality.

## Introduction

Domestic violence and assault (DVA) against women is a fundamental violation of women’s human rights, health, and wellbeing ^1^. Globally, intimate partner violence (IPV) is the most common form of violence against women ^1^. A recent study involving women from marginalised groups in Bangalore city based in southern India reported that over 50% of women had experienced physical domestic violence ever, and 27% faced physical violence in the past six months ^2^. The majority of female victims are married to the perpetrator and underreporting of IPV is a known phenomenon ^3, 4^. IPV is linked to a range of factors including alcohol addiction, financial debt, cultural and social acceptance of violence, and childhood trauma/exposure to violence ^4^. These factors are also linked to gender-based discrimination, adversely affecting girls’ and women’s health and well-being, their ability to continue their education, choose a career, make informed reproductive decisions, and achieve financial independence. Adverse effects of gender-based discrimination on girls’ and women’s health and wellbeing are particularly high in deprived communities ^3, 4^.

Although the need to reduce DVA and empower women in India is widely recognised, there is very limited information available on gender equality-related knowledge, attitudes, and behaviours among Indian adolescents, who are an important age group to target with specific interventions. Interventions to reduce DVA were primarily focused on women for a long-time, and men were not involved in such intervention initiatives. This results in a lack of awareness in men who are often perpetrators subjecting risks to interventions targeted to reduce DVA. However, creating such awareness among men is challenging as it requires change in their attitudes, behaviours by empowering them with knowledge on the importance of gender equality and also on negative impacts of DVA on women and children. Development work to initiate such change requires challenging existing social norms, which come from centuries-old cultural practices, where discrimination against women/girls caused gender-based allocation of resources, work and opportunities across lifespan. Therefore, involvement of men in gender equality related work broadly aims to initiate and sustain a change at individual, family and community level to improve health and wellbeing of women. There is very limited research on the effectiveness of DVA reduction interventions especially from *rural and difficult to access* communities. In India, men engagement expanded in the recent decade thus evaluating such programmes are imperative to inform future work.

With financial and technical support from the SWISSAID ^5^, Halo Medical Foundation (HMF), an NGO working in the Maharashtra state of India, has developed and is currently implementing the ‘Responsible Couples’ intervention to address DVA against women by educating men, supporting women, and providing village resources to create healthy relationships and violence-free communities ^6^. The ‘DEVELOP’ project seeks to increase understanding of gender equality-related knowledge, attitudes, and behaviours among Indian adolescents, as well as evaluate the ‘Responsible Couples’ intervention. The project is planned to be conducted in the Maharashtra state of India in 2019. This paper is a study protocol of the DEVELOP providing detailed information on study objectives, research methods of two main components of the project, data storage, handling, and dissemination plans.

## Protocol

DEVELOP project is designed primarily to support new research collaborations by generating evidence mainly from rural areas of the Maharashtra state of India. The evidence generation is proposed by two key objectives which fit under the overarching project goal as explained below. Research work will involve local staff working full-time at HMF (NGO partner) who will be trained to develop their research capacities and will be engaged in data collection work. Research undertaken through both objectives by a local team will provide them ‘real world’ experiences supporting their future career development. Secondly, our two key partners (SWISSAID and HMF) will benefit from such evidence to inform their on-going as well as forthcoming projects on gender equality in India. The first objective will help our key partners plan projects involving young people through their future expansion, and the second objective will contribute to improve their on-going intervention approach. SWISSAID works with several Indian NGOs, thus findings will be useful in other areas of Maharashtra and nationally in India to design gender equality related work. Research findings, capacity building initiative and collaboration work will also offer valuable experiences for all partners involved to plan future initiatives. The proposed structure helped design the project in line with our funding requirements where local capacity building and research goals are incorporated into this model.

### Project goal

To conduct feasibility and capacity building work in India to support future research and development projects in gender equality.

### Research objectives

- To develop a survey tool and measure gender equality related knowledge, attitudes, and behaviours in Indian adolescents using this tool in rural populations of the Maharashtra state of India.
- To conduct a qualitative evaluation of the current DVA reduction intervention–the ‘Responsible Couples’ project implemented by HMF in rural areas of the Maharashtra state of India.

### Study design

**Figure 1.**
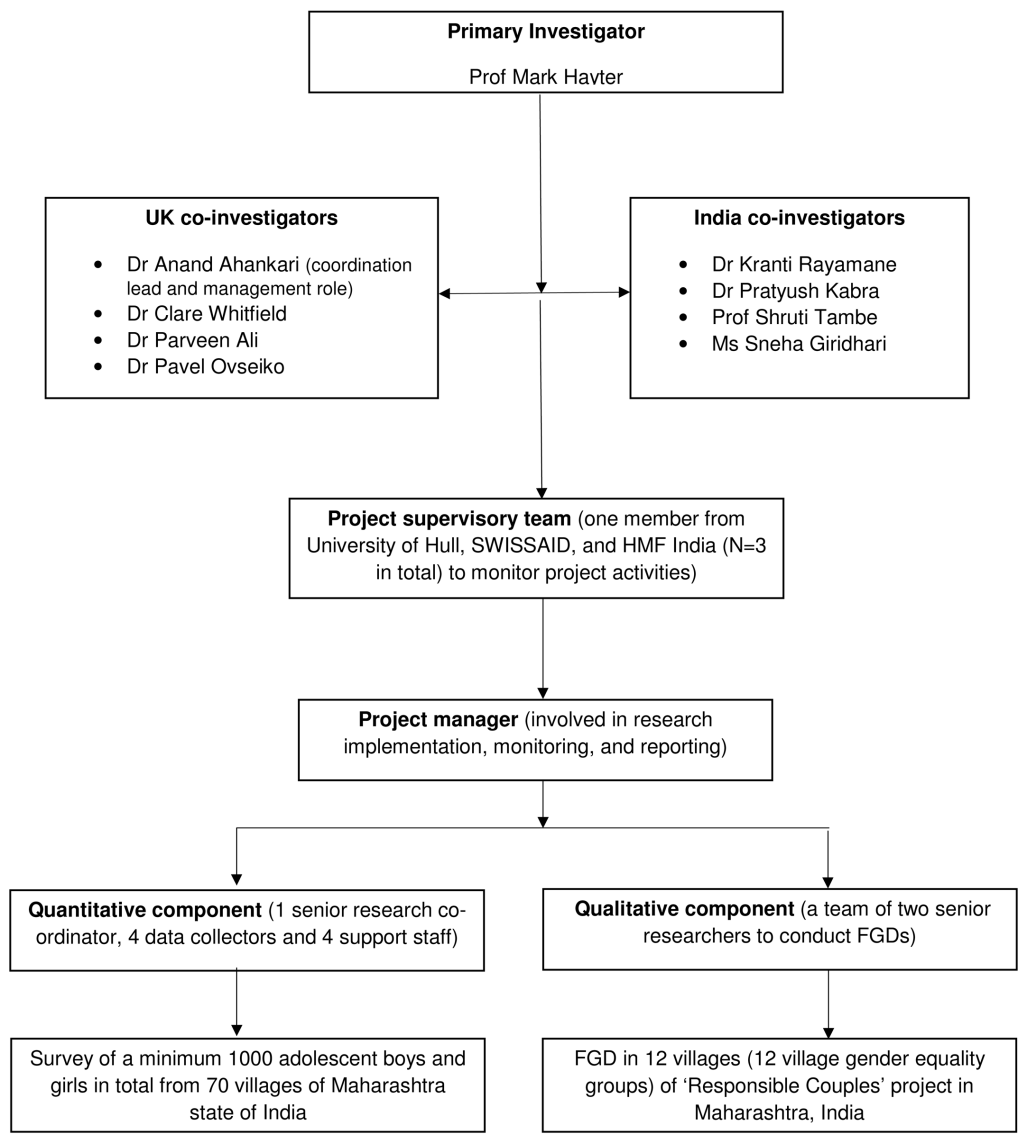
DEVELOP project structure. HMF, Halo Medical Foundation; FGD, focus group discussion.

This study has two components as outlined below to achieve the research objectives. The project team structure and partners are outlined in Figure 1.

I. Quantitative component *-* a questionnaire survey of the gender equality beliefs and attitudes of minimum 1000 young people (male and female) aged 16 to 19 from 70 villages of Maharashtra state, India.

II. Qualitative component *-* focus group discussions with the local gender equality promotion groups in 12 villages of Maharashtra state to explore their views on the implementation of the ‘Responsible Couples’ project- what challenges they are experiencing in their gender equality work, what they feel about the impact of their work, and how the project can be improved further.

I. Quantitative component

### Selection and recruitment

 The study area consists of 70 villages from HMF’s project field, located in the Osmanabad district of Maharashtra state of India. HMF accelerated its programmes in this region following the 1993 earthquake to support emergency relief activities. This geographic area is known for limited healthcare facilities, poor infrastructure, and is also among marginalised regions nationally. Therefore, HMF’s work focus has been in this region over 25 years where various welfare, education, health and development projects are regularly implemented. Majority of the population in the Osmanabad district is in rural areas (approximately 83% rural and 17% urban), and has about 733 villages in total across its 8 blocks. The proposed 70 villages are from two blocks namely Tuljapur and Lohara. The total district population is about 1.6 million and our study area has about 120–140,000 people across 70 villages.

The study will invite all adolescents aged 16 to 19 from 70 villages to participate in the project by completing a questionnaire. These villages define HMF’s current geographic scope based on funding allocated by the SWISSAID to work in Maharashtra. Future programme and intervention development work will involve the same villages; therefore, these were considered in the DEVELOP research project. Each village has a member of HMF staff linked to its programme activities. This individual will help distribute written information about the project (in the form of a leaflet, Supplementary File 1, *Extended data* ^7^) in each village at least two weeks before any data is collected. Leaflets will be distributed to cover all community areas in each village. There are also field staff such as healthcare workers in project area. These workers are also able to provide any verbal description of the project if required at this point in time. They will also orally inform of the date of the data collection event. All staff members including field staff received necessary research and ethics training in February and April 2019 and are supervised by a senior research co-ordinator on a daily basis with additional support from a project manager based at HMF.

Data collectors will then visit the village for one day of data collection. They will set up their station at a village health centre, school or other locally available building/facility, where interested adolescents will be invited to visit to find out more about the project. Written and verbal explanations will be given to those who have not seen the previous information sheet (Supplementary 1, *Extended data*) ^7^.

The study will be open to both boys and girls aged 16 to 19 only. Information on this age related eligibility will be shared through staff trainings including field level support personnel who will ensure that this is shared correctly with potential eligible participants. Self-reported age will be verified verbally on the day of data collection by members of the local research team. Age requirement is mentioned on the participant information sheet which is provided in advance to eligible candidates (Supplementary 1, *Extended data*) ^7^. Participants will be asked to report their age on the data collection form. Participants should be able to read and write in local language (Marathi) in order to understand the project information sheet and complete the data collection form independently. We acknowledge that those who may not be able to read and write will not be eligible to participate in our study. However, considering our ethics processes and confidentiality needs to report gender equality related response with necessary privacy, self-reported approach is preferred. Data collection documents were translated from English to Marathi by a project manager in the first instance, and the translation was verified by authors with bilingual proficiency (AA and SG). The final data files were reviewed several times to ensure its accuracy. The adolescents who agree to participate will be given a questionnaire, pen, and sealable envelope. The on-site data collection staff will address any queries, if asked by participants. There is no financial incentive provided to study participants to avoid any possible coercion.

### Sample size

The adolescent survey will be conducted to collect a minimum of 1000 questionnaires. This number is based on discussions with project partners to ensure that study is deliverable in given resources and time. Research findings may be applicable to wider areas of rural Maharashtra state. The proposed target of 1000 is set considering three months will be available to collect data, however if permitted higher sample size will be achieved depending on project progress. A similar strategy was used to conduct research in this field area involving adolescent girls ^8^. In order to collect representative data by age and gender, a stratified sampling effort will be undertaken. In total, a minimum 1000 questionnaires will be completed with an aim to collect 125 from each age group (16,17,18,19 years), and 500 from each gender. This is the ideal sample scenario; however, no participants will be turned away on a data collection day even if the said number has already been achieved. This strategy is proposed to work towards attaining similar numbers of participants across all age groups to conduct subgroup analysis, if permitted. Any limitations arising from our research design and data observations will be reported along with research findings.

### Survey instrument

A survey questionnaire in the local language (Marathi) will be used to collect data from adolescents. An English version of the questionnaire is available as *Extended data* ^7^. The tool is developed based on a validated and published questionnaire ^9, 10^, which was used to study gender equality among Indian adolescents ^9^. The questionnaire was iteratively revised and improved within the team, discussed with partners, and then piloted and validated prior to administration. The outlined process was completed through focus group discussions and a testing phase involving adolescent boys and girls at the HMF training centre in March 2019. The feedback from the discussions was included, and minor changes were made mainly on the structure of the data collection form. No major changes such as question re-structuring were required. The questionnaire used in this study has a section on basic demographics (12 questions) and then three individual sections to measure knowledge (nine statements), attitudes (six statements) and behaviours (seven statements) related to gender equality (Supplementary 2 *, Extended data*) ^7^. The gender equality score for each participant will be calculated for the three sections of the questionnaire (knowledge, attitudes and behaviour) using the following method. For each statement, the score will range from zero to two. Those who agreed with a given statement, indicating a lack of support for gender inequality, receive a score of zero. Those who partially agree receive a score of one, and those who disagreed receive a score of two, indicating support for gender equality. The total score will be calculated for each completed questionnaire by adding the score for all 22 statements. Total scores for each questionnaire will range from a low of zero (highly gender inequitable) to a high of 44 (highly gender equitable).

### Data collection, analysis and storage

To ensure confidentiality, questionnaires will be completed in an area of the village hall/health centre that affords privacy. The completed questionnaires (in sealed envelopes) are then placed in a box by participants as they leave the hall/data collection centre/station. The overall data collection will be supervised by a qualified member of HMF staff.

The questionnaire will not collect any personal identifiable information such as name, home address, or contact details. Both the study information sheet and the questionnaire will include information assuring the participants of confidentiality and how the data will be used. Participants will not be identified or identifiable through reports or publications and only the research team will have access to the data. All data in India will be stored on a password-protected computers and encrypted USB devices and will only be accessible to the project and research teams. The survey data will be stored on the University of Hull’s secure server and used for analysis purposes. A member(s) of the research team will access the data stored at HMF office in person and will upload the data remotely to the University of Hull’s online storage server using secured login details. This will be verified by another team member to ensure that all data are safely moved to the University of Hull online storage system. The data will be stored for a minimum of five years following the project completion, and will be maintained with lead researcher(s) for future research purposes.

Incomplete questionnaires will be discarded from analysis and stored for auditing purposes. Details on such process including data validation steps will be provided in study methods/results. Survey responses will be analysed in Stata (StataCorp, College Station, Texas, USA) and/or SPSS (IBM) using descriptive statistics, tests of statistical significance, and reliability coefficients. Summary of all collected data will be presented through frequency and percentages for findings from the gender equality tool and research participant demographics. Cronbach’s alpha score for the gender equality scale will be provided. The gender equality tool will be used to calculate a total score for each participant, and will be also used to report overall observations on our study population. This score will be used as a continuous outcome of interest for linear regression purposes. In such analysis, data on individual sociodemographic parameters will be used as an independent exposure variables. Regression analysis will be adjusted depending on availability of data, statistical guidance and published evidence. If data permit, then additional analysis such as logistic regression will be conducted along with comparing results across villages/blocks. Results will be reported in line with STROBE guidelines ^11^, and will be submitted for a peer review publication.

II. Qualitative component

### Selection and recruitment

The ‘Responsible Couples’ project is currently (in 2019) being implemented in 40 villages of Osmanabad district of Maharashtra state, India. These 40 villages are from the wider 70 village network outlined earlier where our NGO partner (HMF) is implementing development work. Each village has one local group comprised of 15 to 20 village members, who are voluntarily working towards gender equality in their community. Group members have been trained by subject experts and receive mentoring support from HMF project implementation staff. The groups provide support and facilitate access to victims of IPV and intervene to prevent violence against women, focussing on those who are married and living with their husband and/or in-laws. Importantly, as part of HMF’s work to support research development, these groups have been involved in the inception of the current research study.

From a list of 40 villages, 12 villages will be randomly selected for focus group discussions. In order to ensure random selection, all villages will be numbered by a project manager based in India, and a total of 12 will be selected by a member of the research team (AA and MH based in the UK). These steps will be completed over email to record the process. At least two weeks before focus group discussions are held, members of the research/project team will convene meetings with the members of the village gender equality groups in the selected villages to describe the project and answer questions. To accompany the verbal description of the project, each group member will receive an information sheet (Supplementary 3, *Extended data*) ^7^. Only existing members of the village gender equality groups in the selected villages will be invited to participate in focus group discussions.

### Sample size

Twelve focus group discussions are expected to provide sufficient insights into the implementation of the ‘Responsible Couples’ intervention across 40 participating villages. It is expected that about 10 members from each village will participate in each focus group discussion. Based on our series of consultations with partners, field visits, interactions with beneficiaries, the proposed 12 focus group discussions is expected to be sufficient to achieve data saturation.

### Focus group discussion instrument

A discussion guide in a local language (Marathi) will be used to facilitate focus group discussions. An English version is included as *Extended data* (Supplementary 4) ^7^. Data collection documents were translated from English to Marathi by a project manager at first instance and were verified by authors with bilingual proficiency (AA and SG). The final data files were reviewed several times to ensure its accuracy. The discussion guide was revised iteratively within the team, discussed with partners, and then piloted prior to research use. The interview guide was used to conduct discussions in two villages from the project areas where its structure, questions were tested. This was attended by a project manager and a senior research co-ordinator to provide feedback to investigators based in India and the UK. No amendments to the guide were required.

### Data collection, analysis and storage

Focus group discussions will be conducted, transcribed, and translated by two experienced facilitators with bilingual skills (Marathi and English) under the supervision of the research team. Efforts will be made to recruit one male and one female researcher for this data collection task to achieve a gender balanced approach. This will provide a comfortable environment for all men and women members/study participants. Further, both researchers will lead on 6 FGD sessions each to provide equal opportunities for skill development through peer and supervisory feedback in line with our capacity development objectives. ** The qualitative data will include information on the views of village level groups on: the implementation of the ‘Responsible Couples’ project; what challenges they are experiencing in their gender equality work; what they feel about the impact of their work; and how the project can be improved further.

No personal information will be collected during the focus groups. The information sheet will include assurances on confidentiality and that no identifiable data will be used in reports and publications. Only the research team will have access to the data. All data in India will be stored on password-protected computers and encrypted USB devices and will only be accessible to the research team. Once research activities are completed in India, the focus group discussion data will be stored on the University of Hull’s system using Microsoft Word and/or PDF files along with the audio recorded discussion files, stored on a secure server and used for analysis purposes. 

Qualitative data from the focus group discussions will be analysed thematically, where two researchers will independently code data and synthesise findings into themes. This will be informed by Braun and Clark’s deductive reasoning methodology ^12^. Additional inputs will be provided by senior qualitative researchers to supervise this process and will also contribute towards such analysis. Furthermore, researchers working on this data will then meet to discuss areas of agreement and disagreement and reach consensus on the coding tree, illustrative quotations, and interpretation. All data findings will be shared with key project partners and data collection to conduct internal peer reviews and checks prior to finalising themes and key findings. Presentation guidelines such as COREQ will be followed wherever deemed necessary ^13^. All agreed themes and sub-themes will be reported in the study results.

### ‘Responsible Couples’ project evaluation

The proposed qualitative data collection through FGD with village level gender equality groups will contribute towards a full evaluation report. The proposed component is preferred during this initial stage of our work (DEVELOP Phase 1, 2018-19) to investigate community/village level change on gender equality related attitudes, behaviors to prevent and reduce DVA against women/girls. The future research (DEVELOP Phase 2, 2020-21) aims to conduct qualitative interviews with service users (men and women) who have used project services offered by village level groups and the NGO partner (HMF). For such future expansion, an independent funding will be sought. Qualitative data from both, service providers and users along with project monitoring reports by our partner organizations will generate evidence towards the final evaluation findings. We acknowledge that evaluating project services only from providers perspective will not be sufficient, thus future initiatives are planned, however such details are not included in this protocol considering it is beyond the current project’s funding and timeframe (DEVELOP Phase 1, 2018-19).

### Ethical statement

The study has been approved by the Faculty of Health Sciences Ethics Committee, University of Hull, UK (approval reference number- FHS125) and the ethics committee of the Ashwini Rural Medical College, Hospital and Research Centre, Solapur, Maharashtra, India (approval reference number- ARMCH/IECHR/03/2019). All survey participants will give individual oral informed consent before completing the questionnaire. The oral informed consent is preferred in this survey to ensure full confidentiality of participating adolescents. Written consent requires basic details such as name, address with further requirements of anonymisation. These details are not requested on the questionnaire and therefore oral informed consent is deemed sufficient from a willing participant before handing over the questionnaire. As a result of this, no personal information on participating adolescents such as name or address will be collected at any point of time. All focus group participants will give a collective written informed consent before participating in focus group discussions. This strategy is decided following consultation with our project partners. At the start of the session, a collective signed consent form will be obtained. In our study area, the provision of a personal signature on a form could be regarded with some suspicion and the collective form alleviates this. Further, all eligible participants are active members of the village level group and regularly meet for monthly meeting and thus are aware about collective signatures as a part of on-going project activities. This also allows the data collection team to collect the signatures of willing participants where individual names, addresses and contact details were not required/collected. The consent form is provided as *Extended data* (Supplementary 5) ^7^.

### Dissemination of information

The results of the study will be disseminated via local, regional, and national dissemination events, online video and blogs, peer-reviewed publications, and presentations at international conferences. A national dissemination workshop will be conducted in Pune, India in July 2019 to share results with NGOs, funding agencies, universities, stakeholders and government representatives. Further, research findings will be shared with communities from 40 villages involved in the qualitative data collection. Such sharing will be coordinated by our NGO partner and will be delivered by members of the research team.

### Study status

The DEVELOP project duration is from December 2018 to July 2019. Research work in India is planned from April to July 2019). This study protocol is revised following peer review in March 2021. The project and data collection activities were completed by 31 ^st^ July 2019. Data analysis work on both components of this study was completed in 2019-20, and two manuscripts developed from this project are being prepared for peer reviewed publications.

## Conclusions

The DEVELOP project will contribute to research capacity building and evidence-based practices in a resource limited setting to achieve our overarching project goal. The project will provide opportunities to train and engage a team of 12 local staff includes data collectors, assistants, researchers in the Maharashtra state of India to improve their knowledge, develop research skills, and enhance experiences of all institutes on international collaborations. It is expected that the project will help partners involved from India and the UK to continue research and also development work on the adoption, implementation, and scale-up of evidence-based gender equality interventions in Maharashtra and other Indian states and territories.

To the best of our knowledge, this will be the first survey from the Maharashtra state of India, and one of the largest surveys, measuring gender equality-related knowledge, attitudes, and behaviours among rural Indian adolescents. The survey findings will generate new valuable insights into how adolescent groups could be engaged in the future to improve gender equality in Indian communities.

The qualitative evaluation will inform the implementation of the ‘Responsible Couples’ intervention and strategies to improve the same through future expansion. It will also have policy implications for HMF, SWISSAID, and other organisations seeking to reduce DVA and empower women in Maharashtra and other parts of the country. Considering diverse Indian culture, practices, and beliefs, the study results should be interpreted carefully beyond the population studied.

## Data availability

### Underlying data

No underlying data are associated with this article.

### Extended data

Figshare: ADolescents GEnder SurVey, REsponsible CoupLes EvaluatiOn, and Capacity Building Project in India (DEVELOP): A study protocol. https://dx.doi.org/10.6084/m9.figshare.8256050.v1 ^7^

This project contains the following extended data:

- Supplementary Files 1 to 5.pdf (participant information sheet for adolescents, questionnaire survey for adolescents in English, participant information sheet for focus group discussion, focus group discussion topic guide in English, focus group discussion consent form)

- DEVELOP_Survey questionnaire in Marathi.pdf (questionnaire in Marathi)

- DEVLOP_FGD Guide in Marathi.pdf (focus group discussion guide in Marathi)

Data are available under the terms of the Creative Commons Zero "No rights reserved" data waiver (CC0 1.0 Public domain dedication).
