# Supplementary material for: aDolescents gEnder surVey, rEsponsible coupLes evaluatiOn, and capacity building Project in India (DEVELOP): a study protocol
Source: F1000Res. 2021 Mar 29;8:958. Originally published 2019 Jun 24. [Version 2] doi: 10.12688/f1000research.19521.2 (PMC8080941; doi:10.12688/f1000research.19521.2)
Supplement: Peer review history

# Peer review: Reviewer response for version 2

**DOI:** 10.5256/f1000research.55438.r82375

**Published:** 2021-04-27

**Authors:** Katharina Goessmann

**Affiliations:** 1 Department of Psychology, Bielefeld University, Bielefeld, 100131, Germany

Thank you to the authors for addressing all my previous queries and for adapting the manuscript accordingly. I approve of all changes made, and would only like to add one thought on the rationale for including men in the efforts to reduce and prevent GBV: Next to a lack of awareness among men, not involving men in those efforts also bears the risk of leaving the responsibility for violence against women with those who experience it. Maybe the authors would like to include this aspect in their rationale.

 Apart from that I am satisfied with the reviewed manuscript and approve of its indexing.

Is the study design appropriate for the research question?

Yes

Is the rationale for, and objectives of, the study clearly described?

Partly

Are sufficient details of the methods provided to allow replication by others?

Partly

Are the datasets clearly presented in a useable and accessible format?

Not applicable

Reviewer Expertise:

partner violence, violence against women, psychotraumatology

I confirm that I have read this submission and believe that I have an appropriate level of expertise to confirm that it is of an acceptable scientific standard.

# Peer review: Reviewer response for version 2

**DOI:** 10.5256/f1000research.55438.r82374

**Published:** 2021-04-07

**Authors:** Prashanth N Srinivas

**Affiliations:** 1 Institute of Public Health, Bengaluru, India

Thank you for your revised version, which addresses all the comments I had raised systematically. My only comment is that when the authors respond about the possible reason for including only those adolescents who can read and write and allude to ethical reasons, another thing to keep in mind is their possible "exclusion". Ethically speaking this too is as much a consideration as is privacy and confidentiality. This consideration must be more comprehensively addressed. The authors assess that this number may be low, but ANY number would still be an exclusion due to the design of the study. Indeed ANY study design may have such in-built exclusions, but this just adds to researcher reflexivity. This is just a comment and does not have a bearing on the overall science and the ethics of the current manuscript.

Is the study design appropriate for the research question?

Yes

Is the rationale for, and objectives of, the study clearly described?

Partly

Are sufficient details of the methods provided to allow replication by others?

Partly

Are the datasets clearly presented in a useable and accessible format?

Not applicable

Reviewer Expertise:

Public health, realist evaluation, health policy & systems research methods

I confirm that I have read this submission and believe that I have an appropriate level of expertise to confirm that it is of an acceptable scientific standard.

# Peer review: Reviewer response for version 1

**DOI:** 10.5256/f1000research.21401.r77963

**Published:** 2021-02-26

**Authors:** Katharina Goessmann

**Affiliations:** 1 Department of Psychology, Bielefeld University, Bielefeld, 100131, Germany

The manuscript, entitled “aDolescents gEnder surVey, rEsponsible coupLes evaluatiOn, and capacity building Project in India (DEVELOP): a study protocol“, presents a study protocol of a proposed study to be conducted within a capacity building project (called DEVELOP) that seeks to plan and perform programs to address gender-based violence (GBV) in India, which is a serious issue with numerous negative consequences, as the authors outline in their introduction.

 The manuscript reports on two components of the project: a quantitative survey on gender-related knowledge, attitudes and practices among adolescents to improve the evidence base in this regard, as well as qualitative evaluation of an ongoing GBV intervention (“Responsible Couples”, RC) in Osmanabad district of Maharashtra, India.

 In addition to those points raised by the first reviewer, I have a few remarks that might help to further improve the manuscript.

**Introduction:**

- From the Title, Abstract and the first part of the introduction, it is not clear whether the study focuses on domestic/partner violence against women, or refers to any kind of GBV against women. Please clarify this from the beginning; also, in the summary of literature from studies reporting GBV globally and in India.
- The authors state that existing intervention programs to reduce GBV have predominantly focused on women. I suggest adding some more information here on why it is important to include men/boys in those efforts.
- It would also be interesting to learn a bit more about the background of the DEVELOP project already in the introduction, such as what are its expected advantages, whether there are other comparable approaches and so on.

**Quantitative component:**

 Selection and recruitment:

  

- The envisaged number of participants in the quantitative survey is 1000. What is the time frame in which you are planning to achieve this number?
- How will the under-age participants give their informed consent? Is the informed consent of parents/guardians required here?

Survey questionnaire (Extended data): In my opinion, the wording of some items could be slightly changed in order to avoid misunderstandings and allow meaningful interpretation:

- Part A, item 5: In its current wording, the item might be understood as asking for the status quo of what “a women’s role” usually is in society or what the adolescents experience in their daily life. Like this, their answer to the item is not necessarily reflective of a lack of support for gender inequality, as it does not directly ask whether the respondent agrees with this widespread societal role of women, or if they think it should be like that. If the item aims to assess the adolescents’ gender beliefs and attitudes, I suggest rephrasing it slightly, for example like this: A woman’s role **should be** taking care of her house and family members / A **real/good** woman’s role is taking care of her house and family members. Similarly, in item 8.
- Part B, item 10: In this item to it is unclear whether it is asking for the adolescents’ opinion/belief in this regard, or for their experience. This should be either rephrased or carefully considered in the interpretation of the data.

**Qualitative component:**

 Data collection:

- Given the sensitivity of the topic, the participants’ gender identities should be considered. Will the focus groups consist of members of all genders or will they be separate for men and women members of the local gender promotion groups? Or are they all female anyway? And what about the gender of the research staff conducting the focus groups?

**Conclusions:**

- The authors state that “the project will provide opportunities to train and engage over 10 researchers and practitioners in Maharashtra state of India to improve their knowledge, develop new research skills, and enhance their experience of collaborating with international partners“. From the described procedures, this expected outcome is not apparent. Please elaborate on what this assumption/aim is based on.

**Minor corrections:**

- Figure 1: the second lowest box on the right should read “Qualitative component”

Is the study design appropriate for the research question?

Yes

Is the rationale for, and objectives of, the study clearly described?

Partly

Are sufficient details of the methods provided to allow replication by others?

Partly

Are the datasets clearly presented in a useable and accessible format?

Not applicable

Reviewer Expertise:

partner violence, violence against women, psychotraumatology

I confirm that I have read this submission and believe that I have an appropriate level of expertise to confirm that it is of an acceptable scientific standard, however I have significant reservations, as outlined above.

# Response

**Published:** 2021-03-11

**Authors:** Anand Ahankari

**Affiliations:** University of Hull, UK

**Response to comments from reviewer 2 (Katharina Goessmann)**

 1. The manuscript, entitled “aDolescents gEnder surVey, rEsponsible coupLes evaluatiOn, and capacity building Project in India (DEVELOP): a study protocol“, presents a study protocol of a proposed study to be conducted within a capacity building project (called DEVELOP) that seeks to plan and perform programs to address gender-based violence (GBV) in India, which is a serious issue with numerous negative consequences, as the authors outline in their introduction. The manuscript reports on two components of the project: a quantitative survey on gender-related knowledge, attitudes and practices among adolescents to improve the evidence base in this regard, as well as qualitative evaluation of an ongoing GBV intervention (“Responsible Couples”, RC) in Osmanabad district of Maharashtra, India.

 In addition to those points raised by the first reviewer, I have a few remarks that might help to further improve the manuscript.

 Response: Thank you for your careful reading of the manuscript and positive feedback. We believe your comments and suggestions have helped us to improve the quality of the manuscript. Please find below our point-by-point responses and revisions.

**2. Introduction:**

 From the Title, Abstract and the first part of the introduction, it is not clear whether the study focuses on domestic/partner violence against women, or refers to any kind of GBV against women. Please clarify this from the beginning; also, in the summary of literature from studies reporting GBV globally and in India.

 Response: We have now revised this across the study protocol by using two key terms- Domestic Violence and Assault (DVA), where intimate partner violence (IPV) is the most common form. Revisions were made to ensure consistency across our writing.

 3. The authors state that existing intervention programs to reduce GBV have predominantly focused on women. I suggest adding some more information here on why it is important to include men/boys in those efforts.

 Response: We have now expanded further on this section as follows.

*Although the need to reduce DVA and empower women in India is widely recognised, there is very limited information available on gender equality-related knowledge, attitudes, and behaviours among Indian adolescents, who are an important age group to target with specific interventions.  Interventions to reduce DVA  were primarily focused on women for a long-time, and men were not involved in  such intervention initiatives. This results in a lack of awareness in men who are often perpetrators subjecting risks to interventions targeted to reduce DVA. However, creating such awareness among men is challenging as it requires change in their attitudes, behaviours by empowering them with knowledge on the importance of gender equality and also on negative impacts of DVA on women and children. Development work to initiate such change requires challenging existing social norms, which come from centuries-old cultural practices, where discrimination against women/girls caused gender-based allocation of resources, work and opportunities across lifespan. Therefore, involvement of men in gender equality related work broadly aims to initiate and sustain a change at individual, family and community level to improve health and wellbeing of women. There is very limited research on the effectiveness of DVA reduction interventions especially from rural and difficult to access communities. In India, men engagement expanded in the recent decade thus evaluating such programmes are imperative to inform future work.*

 4. It would also be interesting to learn a bit more about the background of the DEVELOP project already in the introduction, such as what are its expected advantages, whether there are other comparable approaches and so on.

 Response: Details of the conceptualisation of the DEVELOP project, its relevance and collaborations are explained at the start of the protocol prior to goals and objectives.

*DEVELOP project is designed primarily to support new research collaborations by generating evidence mainly from rural areas of the Maharashtra state of India. The evidence generation is proposed by two key objectives which fit under the overarching project goal as explained below. Research work will involve local staff working full-time at HMF (NGO partner) who will be trained to develop their research capacities and will be engaged in data collection work. Research undertaken through both objectives by a local team will provide them ‘real world’ experiences supporting their future career development. Secondly, our two key partners (SWISSAID and HMF) will benefit from such evidence to inform their on-going as well as forthcoming projects on gender equality in India. The first objective will help our key partners plan projects involving young people through their future expansion, and the second objective will contribute to improve their on-going intervention approach. SWISSAID works with several Indian NGOs, thus findings will be useful in other areas of Maharashtra and nationally in India to design gender equality related work. Research findings, capacity building initiative and collaboration work will also offer valuable experiences for all partners involved to plan future initiatives. The proposed structure helped design the project in line with our funding requirements where local capacity building and research goals are incorporated into this model.*

**5. Quantitative component:**

 Selection and recruitment:

 The envisaged number of participants in the quantitative survey is 1000. What is the time frame in which you are planning to achieve this number?

 Response: We have revised these details as mentioned earlier in this letter in response to the first reviewer and also addressed your question. The revision is outlined below.

*The adolescent survey will be conducted to collect a minimum of 1000 questionnaires. This number is based on discussions with project partners to ensure that study is deliverable in given resources and time. Research findings may be applicable to wider areas of rural Maharashtra state. The proposed target of 1000 is set considering three months will be available to collect data, however if permitted higher sample size will be achieved depending on project progress. A similar strategy was used to conduct research in this field area involving adolescent girls [8]. In order to collect representative data by age and gender, a stratified sampling effort will be undertaken. In total, a minimum 1000 questionnaires will be completed with an aim to collect 125 from each age group (16,17,18,19 years), and 500 from each gender. This is the ideal sample scenario; however, no participants will be turned away on a data collection day even if the said number has already been achieved. This strategy is proposed to work towards attaining similar numbers of participants across all age groups to conduct subgroup analysis, if permitted. Any limitations arising from our research design and data observations will be reported along with research findings.*

 6. How will the under-age participants give their informed consent? Is the informed consent of parents/guardians required here?

 Response: Considering our ethics approval, adolescents over 16 years were permitted to provide consent for their own participation. Furthermore, bearing in mind local ethics requirements in India, eligible participant were verbally informed about the project and were also given participant information sheet to take home and decide on their participation following discussions with their local guardian/parents. There was no financial incentive provided to avoid any possible coercion. No personal identifiable information was required or ever collected from participants where self-reported questionnaire strategy was used.

 7. Survey questionnaire (Extended data): In my opinion, the wording of some items could be slightly changed in order to avoid misunderstandings and allow meaningful interpretation:

 Part A, item 5: In its current wording, the item might be understood as asking for the status quo of what “a women’s role” usually is in society or what the adolescents experience in their daily life. Like this, their answer to the item is not necessarily reflective of a lack of support for gender inequality, as it does not directly ask whether the respondent agrees with this widespread societal role of women, or if they think it should be like that. If the item aims to assess the adolescents’ gender beliefs and attitudes, I suggest rephrasing it slightly, for example like this: A woman’s role **should be** taking care of her house and family members / A **real/good** woman’s role is taking care of her house and family members. Similarly, in item 8 Part B, item 10: In this item to it is unclear whether it is asking for the adolescents’ opinion/belief in this regard, or for their experience. This should be either rephrased or carefully considered in the interpretation of the data.

 Response: We prepared questions based on published literature on this subject, followed by discussions among team members, and then piloted involving adolescents from our study region. This process helped us identify issues around how certain questions were framed and those were revised. Further, advice on providing examples on some questions was implemented carefully. Nevertheless, we acknowledge that the survey tool could be further improved as you have explained. Due to the timeline of our project, data collection and analysis activities are now completed. However, we highly appreciate your advice, which is certainly very useful during future development of our survey tool. We have noted your guidance carefully to make further improvements and then aim to again pilot our survey tool in both rural and urban populations in India for future projects.

**8. Qualitative component:**

 Data collection:

 Given the sensitivity of the topic, the participants’ gender identities should be considered. Will the focus groups consist of members of all genders or will they be separate for men and women members of the local gender promotion groups? Or are they all female anyway? And what about the gender of the research staff conducting the focus groups?

 Response: This is indeed a very important area where project team discussed in detail during research design. All men and women work together through village level gender equality groups, and were also trained jointly as a team by the NGO partner over two years of period. Thus, all FGD will involve men and women during discussions. Two qualitative researchers collected these data having a combination of 1 male and 1 female researcher. As this was possible following a successful recruitment only, thus was not mentioned in this protocol. Nonetheless, we have now included following information to state this intention.

*Focus group discussions will be conducted, transcribed, and translated by two experienced facilitators with bilingual skills (Marathi and English) under the supervision of the research team. Efforts will be made to recruit one male and one female researcher for this data collection task to achieve a gender balanced approach. This will provide a comfortable environment for all men and women members/study participants. Further, both researchers will lead on 6 FGD sessions each to provide equal opportunities for skill development through peer and supervisory feedback in line with our capacity development objectives.*

**9. Conclusions:**

 The authors state that “the project will provide opportunities to train and engage over 10 researchers and practitioners in Maharashtra state of India to improve their knowledge, develop new research skills, and enhance their experience of collaborating with international partners“. From the described procedures, this expected outcome is not apparent. Please elaborate on what this assumption/aim is based on.

 Response: We have revised this information and also linked with the project development and goal where capacity building initiative was mentioned. The revised conclusion is as follows.

*The DEVELOP project will contribute to research capacity building and evidence-based practices in a resource limited setting to achieve our overarching project goal. The project will provide opportunities to train and engage a team of 12 local staff  includes data collectors, assistants, researchers in the Maharashtra state of India to improve their knowledge, develop research skills, and enhance experiences of all institutes on international collaborations. It is expected that the project will help partners involved from India and the UK to continue research and also development work on the adoption, implementation, and scale-up of evidence-based gender equality interventions in Maharashtra and other Indian states and territories.*

*To the best of our knowledge, this will be the first survey from the Maharashtra state of India, and one of the largest surveys, measuring gender equality-related knowledge, attitudes, and behaviours among rural Indian adolescents. The survey findings will generate new valuable insights into how adolescent groups could be engaged in the future to improve gender equality in Indian communities.*

*The qualitative evaluation will inform the implementation of the ‘Responsible Couples’ intervention and strategies to improve the same through future expansion. It will also have policy implications for HMF, SWISSAID, and other organisations seeking to reduce DVA and empower women in Maharashtra and other parts of the country. Considering diverse Indian culture, practices, and beliefs, the study results should be interpreted carefully beyond the population studied.*

**Minor corrections:**

 10. Figure 1: the second lowest box on the right should read “Qualitative component”

 Response: Thank you for noting this error. We have now requested to change this to ‘ **Qualitative’**. A figure indicating this change is also attached with the revised submission and submitted to the F1000.

# Peer review: Reviewer response for version 1

**DOI:** 10.5256/f1000research.21401.r53221

**Published:** 2019-09-16

**Authors:** Prashanth N Srinivas

**Affiliations:** 1 Institute of Public Health, Bengaluru, India

The paper is a study protocol of research proposed in Osmanabad district of Maharashtra, India to improve evidence-base for understanding and acting upon gender-based violence (GBV). The study seeks to understand gender equality related knowledge, attitudes and practices among adolescents on one hand, and evaluate an ongoing intervention to reduce gender-based violence, called “Responsible Couples” (RC). The authors make a strong case for the need for such evidence (from adolescents, where interventions targeting the problem ought to begin) as well as the relative lack of effectiveness evidence from interventions to reduce GBV. Authors indicate that this could be the first of its kind large survey on this topic in Maharashtra, and perhaps among Indian adolescents. In addition, their remarks on the research capacity-building and academic exchange involved are also noted with appreciation. 

**Background**

 The background provides a published estimate of domestic violence (DV) ever and DV in the past 6 months. The study cited surveyed households in one city of south India, whereas the background states this as being an estimate for south India, which may not be an appropriate representation of the original study’s findings. 

**Improving coherence through more contextual information and clarifying objectives**

 A project goal is separately provided in addition to the research objectives. The two research objectives focus on (1) survey tool development for adolescents, and (2) evaluation of the RC program. The project goal is quite broad and both objectives fit within this goal, but it is unclear how these two objectives come together. Is there any specific reason these two objectives have been chosen? What was the rationale for choosing this particular intervention? Perhaps this is related to specific contextual information that may not be part of the protocol. Researchers could consider a section that describes the particular setting where the study is being planned as well as perhaps a section that describes work leading up to the study to provide the reader an understanding of the logic/reasoning in choosing these two objectives in order to work towards the project goal. 

 While the background indicates that the authors will undertake a survey among adolescents (and indeed the data collection and subsequent activities confirm this, the first of the two objectives that include this appears to stop short at “…inform(ing) the development of a survey tool”. This gives an impression of this being a tool-development/feasibility study only. Clarify. 

**Selection and recruitment**

- The project identifies 70 villages which correspond to the area of work of partner NGO as being the study area. It is unclear from the information provided how much of the district this covers, and what the original logic is for the partner NGO to choose these villages. Since a survey is being planned, the reporting of its findings will depend on the overall choice of study site and sampling and hence further information has to be provided to enable an understanding of the study area. Some information on the larger context in terms of how this district compares with other districts in the state with respect to GBV are useful (if available). An estimate of what proportion of the district is covered by these villages is also useful. 
- Authors indicate that the age of eligible respondents for the survey shall be 16 to 19. How will this be verified (if at all)? Will this be based on self-reporting of age? Or will researchers have an estimate/list from prior work? 
- Will estimates/identity of adolescents in these villages be available to researchers from the existing work of partner NGO? If yes, specify how this data will be dealt with and used/not used. Authors may consider comparing their village-wise sample with adolescents in the village to come up with an estimate of their overall survey coverage among adolescents. 
- If adolescents who are unable to read/write turn up, what will the team do? There are both technical and ethical implications of this decision to exclude this group, even if the numbers in this group are small. More so given that educational status has been declared in the background to influence GBV.  

**Sample size**

- Authors state that one of the reasons for choosing a sample size of 1000 is “…to ensure that results from this study will be applicable to rural areas of Maharashtra state.”. This will need to be explained clearly. Specifically, the concern is with respect to (a) how would any sample size defined within a set of villages in one district of the state “…be applicable” to the entire state? Perhaps the authors seek to achieve analytical generalisability of their findings through mixed-method/qualitative inquiry and demonstrate the relative similarity of few/many/all other districts to this district with respect to the area of inquiry, but this cannot be based on a particular sample size in my assessment. It is also noted that another logic to arrive at the size has been the resource availability. On the contrary, could an effort at achieving a sampling strategy of smaller numbers of household visits be used to further minimize sample numbers if needed? I understand that the work may have already begun, in which case, authors need to clarify this section appropriately. 
- Authors mention a stratified sampling approach, but the survey is being conducted by invitation to a “station” at each village. How will the 125 per each age-stratum and 500 from each gender be achieved if there is a patterning in age of children who turn up at the station (for instance, older boys being less “shy” of such a station, or for instance if some stations for reasons unknown are not acceptable/accessible for particular age/gender respondents). Given that the researchers do not identify specific strategies to ensure the sample across strata, this stratified sampling appears more to be a desire at ensuring distribution of sample across all age-groups rather than an actual sampling strategy? Clarify. 

**RC program evaluation**

- In terms of improving the coherence between these 2 objectives, and also given that both are being implemented in the same district, it is useful to understand if the 40 villages where RC will be evaluated are a sub-set of the 70 where the quantitative survey will be conducted?
- The RC group in each village: Are these volunteers or paid staff of the project? Clarify. 
- Given that the objective is to evaluate an intervention which has multiple components, an overall evaluation approach to the intervention is missing. Partly, this could be dealt with by providing more details of how the qualitative data will be analyzed from the FGDs. Also to some extent, the evaluation will only engage with relative insiders (the village level RC group) and will not include perspectives of those who "received" the intervention, a limitation. Authors could consider including a clear schematic/narrative on how the intervention is expected to work outlining the various intervention inputs, assumptions being made and how it is linked to expected outputs of the intervention (akin to a theory of change). In the lack of such a theory, the qualitative data gathered may be difficult to coherently analyze. That said, if there is a plan for doing this in another way, authors could include that. 

**Data analysis**

 There is limited information on how quantitative and qualitative data will be analyzed. This will have implications on the data collection methods and sampling details proposed. Also, see above about evaluation component and (lack of) details of the analysis proposed.

Is the study design appropriate for the research question?

Yes

Is the rationale for, and objectives of, the study clearly described?

Partly

Are sufficient details of the methods provided to allow replication by others?

Partly

Are the datasets clearly presented in a useable and accessible format?

Not applicable

Reviewer Expertise:

Public health, realist evaluation, health policy & systems research methods

I confirm that I have read this submission and believe that I have an appropriate level of expertise to confirm that it is of an acceptable scientific standard, however I have significant reservations, as outlined above.

# Response

**Published:** 2021-03-11

**Authors:** Anand Ahankari

**Affiliations:** University of Hull, UK

**Response to comments from reviewer 1 (Prashanth N Srinivas)**

 1. The paper is a study protocol of research proposed in Osmanabad district of Maharashtra, India to improve evidence-base for understanding and acting upon gender-based violence (GBV). The study seeks to understand gender equality related knowledge, attitudes and practices among adolescents on one hand, and evaluate an ongoing intervention to reduce gender-based violence, called “Responsible Couples” (RC). The authors make a strong case for the need for such evidence (from adolescents, where interventions targeting the problem ought to begin) as well as the relative lack of effectiveness evidence from interventions to reduce GBV. Authors indicate that this could be the first of its kind large survey on this topic in Maharashtra, and perhaps among Indian adolescents. In addition, their remarks on the research capacity-building and academic exchange involved are also noted with appreciation. 

 Response: Thank you for your careful reading of the manuscript and positive feedback. We believe your comments and suggestions have helped us to improve the quality of the manuscript. Please find below our point-by-point responses and revisions.

**2. Background**

 The background provides a published estimate of domestic violence (DV) ever and DV in the past 6 months. The study cited surveyed households in one city of south India, whereas the background states this as being an estimate for south India, which may not be an appropriate representation of the original study’s findings. 

 Response: We have revised the introduction/background as follows.

*A recent study involving women from marginalised groups in Bangalore city based in southern India reported that over 50% of women had experienced physical domestic violence ever, and 27% faced physical  violence in the past six months* *^2^* ** *.*

**3. Improving coherence through more contextual information and clarifying objectives**

 A project goal is separately provided in addition to the research objectives. The two research objectives focus on (1) survey tool development for adolescents, and (2) evaluation of the RC program. The project goal is quite broad and both objectives fit within this goal, but it is unclear how these two objectives come together. Is there any specific reason these two objectives have been chosen? What was the rationale for choosing this particular intervention? Perhaps this is related to specific contextual information that may not be part of the protocol. Researchers could consider a section that describes the particular setting where the study is being planned as well as perhaps a section that describes work leading up to the study to provide the reader an understanding of the logic/reasoning in choosing these two objectives in order to work towards the project goal. 

 Response: We have provided a more detailed explanation.  

*DEVELOP project is designed primarily to support new research collaborations by generating evidence mainly from rural areas of the Maharashtra state of India. The evidence generation is proposed by two key objectives which fit under the overarching project goal as explained below. Research work will involve local staff working full-time at HMF (NGO partner) who will be trained to develop their research capacities and will be engaged in data collection work. Research undertaken through both objectives by a local team will provide them ‘real world’ experiences supporting their future career development. Secondly, our two key partners (SWISSAID and HMF) will benefit from such evidence to inform their on-going as well as forthcoming projects on gender equality in India. The first objective will help our key partners plan projects involving young people through their future expansion, and the second objective will contribute to improve their on-going intervention approach. SWISSAID works with several Indian NGOs, thus findings will be useful in other areas of Maharashtra and nationally in India to design gender equality related work. Research findings, capacity building initiative and collaboration work will also offer valuable experiences for all partners involved to plan future initiatives. The proposed structure helped design the project in line with our funding requirements where local capacity building and research goals are incorporated into this model.*

 4. While the background indicates that the authors will undertake a survey among adolescents (and indeed the data collection and subsequent activities confirm this, the first of the two objectives that include this appears to stop short at “…inform(ing) the development of a survey tool”. This gives an impression of this being a tool-development/feasibility study only. Clarify. 

 Response: We have revised objectives to improve clarity and ensure that survey tool development and its proposed use is reflected with study location.

*To develop a survey tool and measure gender equality related knowledge, attitudes, and behaviours in Indian adolescents using this tool in rural populations of the Maharashtra state of India.*

*To conduct a qualitative evaluation of the current DVA reduction intervention–the ‘Responsible Couples’ project implemented by HMF in  rural areas of the Maharashtra state of India.*

**5. Selection and recruitment**

 The project identifies 70 villages which correspond to the area of work of partner NGO as being the study area. It is unclear from the information provided how much of the district this covers, and what the original logic is for the partner NGO to choose these villages. Since a survey is being planned, the reporting of its findings will depend on the overall choice of study site and sampling and hence further information has to be provided to enable an understanding of the study area. Some information on the larger context in terms of how this district compares with other districts in the state with respect to GBV are useful (if available). An estimate of what proportion of the district is covered by these villages is also useful. 

 Response: To the best of our knowledge, there are no published studies from this region to report, but we have provided information on the study area and connected this further with NGO’s work. 

    

*The study area consists of 70 villages from HMF’s project field, located in the Osmanabad district of Maharashtra state of India. HMF accelerated its programmes in this region following the 1993 earthquake to support emergency relief activities. This geographic area is known for limited healthcare facilities, poor infrastructure, and is also among marginalised regions nationally. Therefore, HMF’s work focus has been in this region over 25 years where various welfare, education, health and development projects are regularly implemented. Majority of the population in the Osmanabad district is in rural areas (approximately 83% rural and 17% urban), and has about 733 villages in total across its 8 blocks. The proposed 70 villages are from two blocks namely Tuljapur and Lohara. The total district population is about 1.6 million and our study area has about 120-140,000 people across 70 villages.*

 6. Authors indicate that the age of eligible respondents for the survey shall be 16 to 19. How will this be verified (if at all)? Will this be based on self-reporting of age? Or will researchers have an estimate/list from prior work? 

 Response: Data collection team will use self-reported age. All project staff were made aware about this requirement and such eligibility information is shared verbally as well as through participant information sheet. 

*The study will be open to both boys and girls aged 16 to 19 only. Information on this age related eligibility will be shared through staff trainings including field level support personnel who will ensure that this is shared correctly with potential eligible participants. Self-reported age will be verified verbally on the day of data collection by members of the local research team. Age requirement is mentioned on the participant information sheet which is provided in advance to eligible candidates (Supplementary 1, Extended data) [7]. Participants will be asked to report their age on the data collection form.*

 7. Will estimates/identity of adolescents in these villages be available to researchers from the existing work of partner NGO? If yes, specify how this data will be dealt with and used/not used. Authors may consider comparing their village-wise sample with adolescents in the village to come up with an estimate of their overall survey coverage among adolescents. 

 Response: Such information is not collected, stored or used anytime during the project due to the ethical implications. At the planning stage of the project, we considered estimating the overall survey coverage, but this was not feasible because unfortunately information on the numbers and socio-demographic characteristics of adolescents by village or region was not available.

 8. If adolescents who are unable to read/write turn up, what will the team do? There are both technical and ethical implications of this decision to exclude this group, even if the numbers in this group are small. More so given that educational status has been declared in the background to influence GBV.  

 Response: Indeed, only those who are able to read and write will be able to participate in this study. This was preferred instead of interview questionnaire to ensure confidentiality and avoid reporting bias. From the experience of our partners and the lead researcher on our team who have previously conducted five research projects in this area, overall literacy skills among adolescents are sufficient to fully understand and participate in the survey. We have acknowledged this limitation in our revised draft.

*We acknowledge that those who may not be able to read and write will not be eligible to participate in our study. However, considering our ethics processes and confidentiality needs to report gender equality related response with necessary privacy, self-reported approach is preferred.*

**9. Sample size**

 Authors state that one of the reasons for choosing a sample size of 1000 is “…to ensure that results from this study will be applicable to rural areas of Maharashtra state.”. This will need to be explained clearly. Specifically, the concern is with respect to (a) how would any sample size defined within a set of villages in one district of the state “…be applicable” to the entire state? Perhaps the authors seek to achieve analytical generalisability of their findings through mixed-method/qualitative inquiry and demonstrate the relative similarity of few/many/all other districts to this district with respect to the area of inquiry, but this cannot be based on a particular sample size in my assessment. It is also noted that another logic to arrive at the size has been the resource availability. On the contrary, could an effort at achieving a sampling strategy of smaller numbers of household visits be used to further minimize sample numbers if needed? I understand that the work may have already begun, in which case, authors need to clarify this section appropriately. Authors mention a stratified sampling approach, but the survey is being conducted by invitation to a “station” at each village. How will the 125 per each age-stratum and 500 from each gender be achieved if there is a patterning in age of children who turn up at the station (for instance, older boys being less “shy” of such a station, or for instance if some stations for reasons unknown are not acceptable/accessible for particular age/gender respondents). Given that the researchers do not identify specific strategies to ensure the sample across strata, this stratified sampling appears more to be a desire at ensuring distribution of sample across all age-groups rather than an actual sampling strategy? Clarify. 

 Response: We have revised this section to improve clarity:

*The adolescent survey will be conducted to collect a minimum of 1000 questionnaires. This number is based on discussions with project partners to ensure that study is deliverable in given resources and time. Research findings may be applicable to wider areas of rural Maharashtra state. The proposed target of 1000 is set considering three months will be available to collect data, however if permitted higher sample size will be achieved depending on project progress. A similar strategy was used to conduct research in this field area involving adolescent girls [8]. In order to collect representative data by age and gender, a stratified sampling effort will be undertaken. In total, a minimum 1000 questionnaires will be completed with an aim to collect 125 from each age group (16,17,18,19 years), and 500 from each gender. This is the ideal sample scenario; however, no participants will be turned away on a data collection day even if the said number has already been achieved. This strategy is proposed to work towards attaining similar numbers of participants across all age groups to conduct subgroup analysis, if permitted. Any limitations arising from our research design and data observations will be reported along with research findings.*

**10. RC program evaluation**

 In terms of improving the coherence between these 2 objectives, and also given that both are being implemented in the same district, it is useful to understand if the 40 villages where RC will be evaluated are a sub-set of the 70 where the quantitative survey will be conducted?

 Response: Yes, the said 40 villages make the total of 70. We have revised the manuscript accordingly.

*The ‘Responsible Couples’ project is currently (in 2019) being implemented in 40 villages of Osmanabad district of Maharashtra state, India. These 40 villages are from the wider 70 village network outlined earlier where our NGO partner (HMF) is implementing development work. Each village has one local group comprised of 15 to 20 village members, who are voluntarily working towards gender equality in their community.*

 11. The RC group in each village: Are these volunteers or paid staff of the project? Clarify. 

 Response: Village level gender equality group members work voluntarily. We have mentioned this in the revised text above.

 12. Given that the objective is to evaluate an intervention which has multiple components, an overall evaluation approach to the intervention is missing. Partly, this could be dealt with by providing more details of how the qualitative data will be analyzed from the FGDs. Also to some extent, the evaluation will only engage with relative insiders (the village level RC group) and will not include perspectives of those who "received" the intervention, a limitation. Authors could consider including a clear schematic/narrative on how the intervention is expected to work outlining the various intervention inputs, assumptions being made and how it is linked to expected outputs of the intervention (akin to a theory of change). In the lack of such a theory, the qualitative data gathered may be difficult to coherently analyze. That said, if there is a plan for doing this in another way, authors could include that. 

 Authors response: We have secured a follow-up funding where qualitative interviews were conducted through the DEVELOP Phase 2 work. This involved in-depth interviews with men and women who utilised project services. Research data collection for this was completed in 2020, and currently this data are being prepared for analysis. We have mentioned this in our evaluation approach which is a new section included in the protocol. 

**‘Responsible Couples’ project evaluation**

*The proposed qualitative data collection through FGD with village level gender equality groups will contribute towards a full evaluation report. The proposed component is preferred during this initial stage of our work (DEVELOP Phase 1, 2018-19) to investigate community/village level change on gender equality related attitudes, behaviors to prevent and reduce DVA against women/girls. The future research (DEVELOP Phase 2, 2020-21) aims to conduct qualitative interviews with service users (men and women) who have used project services offered by village level groups and the NGO partner (HMF). For such future expansion, an independent funding will be sought. Qualitative data from both, service providers and users along with project monitoring reports by our partner organizations will generate evidence towards the final evaluation findings. We acknowledge that evaluating project services only from providers perspective will not be sufficient, thus future initiatives are planned, however such details are not included in this protocol considering it is beyond the current project’s funding and timeframe (DEVELOP Phase 1, 2018-19).*

**13. Data analysis**

 There is limited information on how quantitative and qualitative data will be analyzed. This will have implications on the data collection methods and sampling details proposed. Also, see above about evaluation component and (lack of) details of the analysis proposed.

 Authors response: We have revised data analysis plan for both qualitative and quantitative sections as follows. 

*For quantitative component*

*Incomplete questionnaires will be discarded from analysis and stored for auditing purposes. Details on such process including data validation steps will be provided in study methods/results. Survey responses will be analysed in Stata (StataCorp, College Station, Texas, USA) and/or SPSS (IBM) using descriptive statistics, tests of statistical significance, and reliability coefficients. Summary of all collected data will be presented through frequency and percentages for findings from the gender equality tool and research participant demographics. Cronbach’s alpha score for the gender equality scale will be provided. The gender equality tool will be used to calculate a total score for each participant, and will be also used to report overall observations from on our study population. This score will be used as a continuous outcome of interest for linear regression purposes. In such analysis, data on individual sociodemographic parameters will be used as an independent exposure variables. Regression analysis will be adjusted depending on availability of data, statistical guidance and published evidence. If data permit, then additional analysis such as logistic regression will be conducted along with comparing results across villages/blocks. Results will be reported in line with STROBE guidelines [11] and will be submitted for a peer review publication.*

*For qualitative component*

*Qualitative data from the focus group discussions will be analysed thematically, where two researchers will independently code data and synthesise  findings into themes. This will be informed by* *Braun and Clark’s deductive reasoning methodology* *^12^* *.* *Additional inputs will be provided by senior qualitative researchers to supervise this process and will also contribute towards such analysis. Furthermore,  researchers working on this data will then meet to discuss areas of agreement and disagreement and reach consensus on the coding tree, illustrative quotations, and interpretation. All data findings will be shared with key project partners and data collection to conduct internal peer reviews and checks prior to finalising themes and key findings. Presentation guidelines such as COREQ will be followed wherever deemed necessary  [13]. All agreed themes and sub-themes will be reported in the study results.*
